# Potent *ex vivo* expanded, human CD34+ cord blood-derived natural killer cells for cancer immunotherapy

**DOI:** 10.1186/2051-1426-3-S2-P62

**Published:** 2015-11-04

**Authors:** Xiaokui Zhang, Lin Kang, Ivana Djuretic, Eric Law, Vanessa Voskinarian-Berse, Jeffrey Harris, Uri Herzberg, Wolfgang Hofgartner, Robert Hariri

**Affiliations:** 1 Celgene Cellular Therapeutics, Warren, NJ, USA

## Background

Clinical studies suggest that adoptive transfer of allogeneic natural killer (NK) cells represent a promising treatment for patients with hematological malignancies and solid tumors. Celgene Cellular Therapeutics has established a cultivation process to generate human NK cells from umbilical cord blood (UCB) CD34^+^ cells with substantial cytolytic activity against several human tumor cell lines.

## Method

UCB CD34^+^ cells were cultivated in presence of cytokines including thromobopoietin, SCF, Flt3 ligand, IL-7, IL-15 and IL-2 for 35 days to produce Placental Intermediate Natural Killer (PiNK) cells. Multi-color flow cytometry was used to determine the phenotypic characteristics of PiNK cells. Cytotoxicity assays were performed by co-culturing PiNK cells with tumor cell lines for 4 hours. Furthermore, supernatants were collected to analyze secreted perforin, granzymes and cytokines.

## Results

Using the cultivation process, a highly pure population (88.3% ± 6.3%) of CD3^-^CD56^+^ NK cells was routinely achieved. PiNK cells display a developmentally intermediate immunophenotype, evidenced by the low / negative expression of CD16 and KIRs. PiNK cells express the natural cytotoxicity receptors (NKp30, NKp46 and NKp44), the c-lectin receptors (CD94, NKG2D and CD161), DNAM-1, 2B4, CD117, and CD11a. Cytolytic mediators (perforin and granzymes) and Eomes, the regulator of NK cell maturation and cytolytic function, were detected in PiNK cells. PiNK cells exhibit cytotoxicity against hematological tumor cell lines *in vitro*. At an effector to target ratio of 10:1, PiNK cells exert lysis towards cell lines, including CML (K562, 70.3% ± 14.8%), AML (HL-60, 31.0% ± 17.8%) and multiple myeloma (RPMI8266, 32.4% ± 19.5%). When co-cultured with K562 cells at a 1:1 ratio for 24 hours, PiNK cells produce functional cytokines including IFN-γ, TNF-α and GM-CSF. Confocal imaging revealed that PiNK cells, when in contact with tumor cells, formed an F-actin immunological synapse with polarization of perforin. Furthermore, in the presence of anti-CD20 (Rituximab, 10 mg/mL), the cytotoxicity of PiNK cells against Daudi cells (Burkitt's lymphoma) increased from 7.3% ± 8.0% to 35.1% ± 5.7%, demonstrating potent antibody-dependent cell-mediated cytotoxicity (ADCC).

## Conclusions

Large quantities of functionally active PiNK cells can be generated from UCB CD34+ progenitors. These cells elicit anti-tumor activity via direct cytolysis, ADCC, and secreted effector cytokines. PiNK cells represent an allogeneic cellular immunotherapy product with potential applications for patients with hematologic malignancies and solid tumors.

